# Systematic Review of Cryptococcus neoformans Seroprevalence, Antifungal Susceptibility, and Pathogenesis in Patients With HIV/AIDS on Combination Antiretroviral Therapy in Abuja, Nigeria

**DOI:** 10.7759/cureus.81698

**Published:** 2025-04-04

**Authors:** Gloria C Ifiora, Ijeje Sule, Edediong Ekarika, Chigozie D Opara, Henry Adebayo

**Affiliations:** 1 Pharmaceutical Microbiology, Nnamdi Azikiwe University, Awka, NGA; 2 Cardiology, Red Deer Regional Hospital Centre, Red Deer, CAN; 3 Public Health, Emory Rollins School of Public Health, Atlanta, USA; 4 Medicine, All Saints University School of Medicine, Roseau, DMA; 5 Medicine and Surgery, Imo State University, Owerri, NGA; 6 Orthodontics, University of Benin, Benin City, NGA

**Keywords:** antiretroviral therapy, combination antiretroviral therapy (cart), cryptococcosis, cryptococcus neoformans (c. neoformans), people living with hiv/aids

## Abstract

For individuals living with HIV/AIDS in low-resource settings such as Sub-Saharan African countries, including Nigeria, cryptococcal meningitis (CM) is a significant contributor to both mortality and morbidity. Despite advancements in antiretroviral therapy (ART), which have markedly transformed the treatment and management of HIV, CM remains a considerable challenge. It primarily arises from delays in diagnosis, limited access to antifungal treatments, and a lack of healthcare resources and infrastructure. With the historical correlation between the rising prevalence of HIV/AIDS and the increased incidence of *Cryptococcus neoformans* infections, understanding the genetic diversity and virulence factors of these pathogens is essential. Sub-Saharan Africa continues to face elevated rates of CM-related deaths. This underscores the urgent need for effective intervention strategies. The objective of this study is to determine the seroprevalence of the *Cryptococcus* species within the Abuja population, evaluate the susceptibility of circulating *C. neoformans *clinical isolates to the antifungal medications widely used for treating cryptococcosis, and assess the correlation between the levels of micronutrients and the progression of *Cryptococcus* infection in individuals with HIV/AIDS. To this end, we conducted an extensive literature search across various online databases, including Embase, PubMed, Scopus, Web of Science, and Google Scholar. The studies analyzed included those with a crossover design, randomized controlled trials (RCTs), systematic reviews, meta-analyses, and prospective cohort studies focused on palliative care in heart failure patients. Fifteen studies were included in accordance with the Preferred Reporting Items for Systematic Reviews and Meta-Analyses (PRISMA) guidelines. The study findings support public health initiatives by informing screening protocols, enhancing treatment regimens, and improving overall patient care outcomes. By advancing the understanding of the pathogenesis and transmission of *C. neoformans*, this study contributes to global efforts aimed at reducing CM-associated morbidity and mortality rates among patients with HIV. In this context, it establishes a seroprevalence baseline for cryptococcosis in Abuja, Nigeria, identifies virulence-associated genetic markers, and recommends integrating cryptococcal screening into HIV care and management.

## Introduction and background

Despite improvements in access to antiretroviral therapy (ART), which have significantly changed the prognosis for individuals living with HIV in resource-limited contexts, treatment coverage remains low. Many individuals are diagnosed with HIV at much later stages of the disease [1]. Additionally, the burden of opportunistic infections continues to pose a considerable challenge to achieving optimal HIV care. In resource-limited settings, patients often succumb to various HIV-related opportunistic infections both before and shortly after starting ART. Recent reports highlight the troubling challenge of cryptococcal meningitis (CM) in Sub-Saharan Africa (SSA), emphasizing that much work remains to be done to improve the diagnosis and management of this condition [2,3].

Cryptococcosis is a rare opportunistic mycosis caused by the encapsulated yeast *Cryptococcus neoformans*. It can present as lethal CM or non-meningeal infections, which include pulmonary infections, cryptococcosis, and cutaneous infections [2-4]. Initially discovered over a century ago from environmental sources [4,5], *C. neoformans* refers to the encapsulated, heterobasidiomycetous fungus that is universally distributed as an infectious opportunistic pathogen, mainly among persons living with HIV. The initial cases involving cryptococcosis in both humans and animals were recorded during the later years of the 19th century, despite their cumulative incidence increasing significantly during the 1900s [6]. In SSA, the cases of cryptococcosis started increasing between 1940 and 1960. This has been widely attributed to the advent of HIV/AIDS within the region of the Congo River basin [6]. Thus, the profile of the genus *Cryptococcus* was globally elevated by the HIV/AIDS epidemic from a simple unknown yeast pathogen to an increasingly important fungal cause of mortality and morbidity [6,7]. The spectrum of clinical infection with *C. neoformans* ranges from the absolute and asymptomatic state to increasingly severe and disseminated disease that could be fatal. The pathogen mainly enters the body via the respiratory tract, even though it has a predilection for the central nervous system (CNS). Generally, *C. neoformans *is not regarded as a part of the normal human flora [5] and is normally isolated in immunocompromised persons, such as patients with HIV/AIDS, individuals on immunosuppressive treatment, patients with neck and head cancer, and those suffering from lymphoproliferative diseases.

Surveys conducted in the United States have predicted an overall annual prevalence of cryptococcosis in the pre-HIV/AIDS period of approximately 0.8 cases per 100,000 persons [8]. Nonetheless, during the peak of the HIV/AIDS epidemic in the country, which occurred during the early 1990s, the annual prevalence of cryptococcosis was approximately five per 100,000 persons across the major cities. The roll-out of the highly active antiretroviral therapy (HAART) in high-income nations, including the United States, during the mid-1990s significantly reduced the overall prevalence of cryptococcosis to nearly its pre-AIDS state [5]. However, in the SSA region, data regarding the prevalence of CM remains limited, although more than 700,000 cases are estimated to be reported annually, leading to over 500,000 deaths [9-11]. Likewise, meningitis due to* C. neoformans* has been acknowledged to occur in approximately 50% of persons with HIV in the SSA as well as in developing nations [10]. The higher case fatality rate has been linked to the absence of effective diagnostic equipment along with inappropriate treatment alternatives in these resource-constrained environments. The higher prevalence of CM infections in the SSA alongside other resource-limited contexts present the various stakeholders with an important opportunity to work jointly towards addressing and tackling the fast-developing CM epidemic [9-12]. Furthermore, HIV/AIDS-related cryptococcal infection is presently responsible for approximately 80% of the global cases of cryptococcal infections [5]. In immunocompromised patients, including persons with HIV/AIDS, infection outcomes with *C. neoformans* are normally fatal. Moreover, those who experience clinical recovery from CM normally require a lasting secondary prophylaxis treatment. Various studies have also indicated that the mortality attributable to CM in persons living with HIV/AIDS ranges between 25% and 30%. Additionally, approximately 40% of individuals who recover from this infection are likely to suffer from neurological sequelae, cranial palsies, hydrocephalus, loss of vision, and forms of mental retardation [5-10]. Moreover, approximately 25% of the cases are prone to a relapse [5-10]. 

To address the high mortality rates, the World Health Organization (WHO) has initiated the 'rapid advice' guidelines for individuals living with HIV/AIDS in low-resource settings like the SSA. Effective reduction of deaths due to CM necessitates early detection. Additionally, among the primary WHO recommendations is to consider cryptococcal antigen (CrAg) screening along with preemptive antifungal treatment for those with a positive HIV test. This is especially recommended in ART-naive adults with a CD4 count below 100 cells/µl and living in regions where cryptococcal disease is prevalent [1,5-11]. This is well-supported by various clinical and epidemiological studies that have revealed a high occurrence of cryptococcal antigenemia in ART-naïve adults in multiple resource-limited settings, along with an increased one-year mortality rate in such patients. Additionally, screening and treating those living with HIV and cryptococcal antigenemia is cost-effective. Furthermore, evidence suggests that cryptococcal antigenemia can occur nearly 22 days prior to the onset of CM, further strengthening the rationale for prompt screening and treatment [5-11].

Reports of cryptococcal microbial infections have been noted in various regions of Nigeria [13-15]. Nevertheless, there is a scarcity of information regarding their prevalence and impact in patients with HIV/AIDS within Abuja, Federal Capital Territory (FCT). The combination of a high rate of HIV along with the potentially elevated prevalence and severity of AIDS and opportunistic infections highlights the necessity for research on the occurrence of cryptococcal infections in this region [13-15]. This investigation has also gained importance due to the major challenges in the early detection of the *Cryptococcus* species, which was previously hindered by inadequate testing methods, particularly blood cultures, and the prohibitive expense of more sophisticated techniques. However, advancements in methods of antigen serology have improved this situation [15].

Many earlier studies have focused on isolating and categorizing *C. neoformans* and *C. gattii *from infected individuals, as well as identifying strains based on their morphological characteristics, primarily in patients with HIV/AIDS and compromised immune systems [14,15]. However, our understanding of the genetic diversity of this significant pathogen remains limited. Thus, characterizing these isolates is crucial for providing foundational data necessary for effective infection control strategies. Additionally, the current knowledge regarding the pathogenic factors of these strains is still scarce. Consequently, this study's objective is to determine the seroprevalence of *Cryptococcus* species within the Abuja population, evaluate the susceptibility of circulating *C. neoformans* clinical isolates to the antifungal medications widely used in cryptococcosis treatment, and assess the correlations between micronutrient levels and progression of cryptococcal infections in persons with HIV/AIDS. The findings from this research will support the development of strategies for the prevention and management of *C. neoformans* infections in Nigeria. Therefore, this study also aims to examine the genomic features of the circulating cryptococcal strains, understand their prevalence and pathogenicity in this region, analyze the distribution of infections based on gender and age, evaluate the influence of occupation on the incidence of cryptococcal infections, and offer valuable information to enhance knowledge about cryptococcosis in the context of HIV for public health improvement.

## Review

Materials and methods

For this systematic review, an in-depth literature search was conducted on various online databases, such as PubMed, Scopus, Web of Sciences, Embase, and Google Scholar, for pertinent studies published between 2010 and 2024. The articles chosen encompassed prospective cohort studies, epidemiological research, systematic reviews, health assessment studies, and multicentric investigations. Duplicate sources were pinpointed by matching studies with similar population years. Additionally, various National Library of Medicine's (NLM) Medical Subject Headings (MeSH) keywords were utilized in the literature search, including "cryptococcosis," "*Cryptococcal neoformans* infection," "HIV/AIDS," "antiretroviral (ART) therapy," and "individuals living with HIV/AIDS." The comprehensive literature search resulted in 1219 articles.

Inclusion and exclusion criteria

The selection of relevant studies, along with the removal of duplicates, was conducted following a three-phase procedure. The initial phase involved reviewing the titles and abstracts of the studies, while the second phase focused on excluding articles deemed irrelevant to this investigation. The final phase required a thorough assessment of the full texts of each study to confirm their relevance to this research. As a result, three independent reviewers were tasked with screening the studies, and any disagreements were resolved through discussion and consensus.

Furthermore, the inclusion criteria aimed at original studies, including randomized controlled trials (RCTs), prospective cohort studies, and those with a crossover design. To qualify under the established criteria, a study must have been published between 2010 and 2024, originally in English, and represent original scientific research published in reputable journals. Additionally, the studies that qualified were those that examined the incidence of cryptococcosis infection in patients with HIV/AIDS in Abuja, Nigeria, as well as in the surrounding regions and countries. Both human and animal studies were included in the analysis. 

Conversely, the exclusion criteria encompassed sponsored clinical trials, editorials, opinion pieces, narrative reviews, and studies not relevant to the target populations. Studies that were inaccessible or irrelevant, as well as those that employed poor methodologies, were also excluded, resulting in the removal of 1,199 articles.

Quality assessment

The studies that were included were assessed using the Appraisal tool for Cross-Sectional Studies (AXIS) [16], a tool for critical appraisal that consists of 20 items for studies. Consequently, all the selected studies were reviewed by three independent evaluators, with any potential disagreements resolved through consensus and discussions. Additionally, each study that was included was assigned a score of 1 (yes) or 0 (no), and "don't know" for items that were not applicable. In general, the quality of the included studies ranged from moderate to high, with six studies classified as moderate quality and the other nine as high quality.

Risk of bias (RoB) assessment

For this systematic review, a RoB was conducted to assess the included the internal validity of the studies. Therefore, the objective of conducting a RoB was to establish if the findings of the included studies were affected by various biases capable of resulting in an under- or overestimation of the actual effects of the association and intervention. The Measurement Tool to Assess Systematic Reviews (AMSTAR) 2 critical appraisal tool was employed in assessing the quality of the studies, including the determination of reporting bias, detection bias, selection bias, and confounding bias [17].

Data extraction

The researchers utilized a data extraction template to gather information from the selected studies. Data extracted from the studies included general attributes such as the names of the authors, research design, sampling techniques, publication year, and demographic details including sample size, age, gender, race, and follow-up information. Additionally, data regarding the interventions applied, the duration of the intervention, and the assessment methods utilized were recorded. The primary findings of each study were also meticulously documented. The three reviewers independently performed the data extraction and any potential disagreements were settled through consensus and discussions among them.

Results

**Figure 1:**
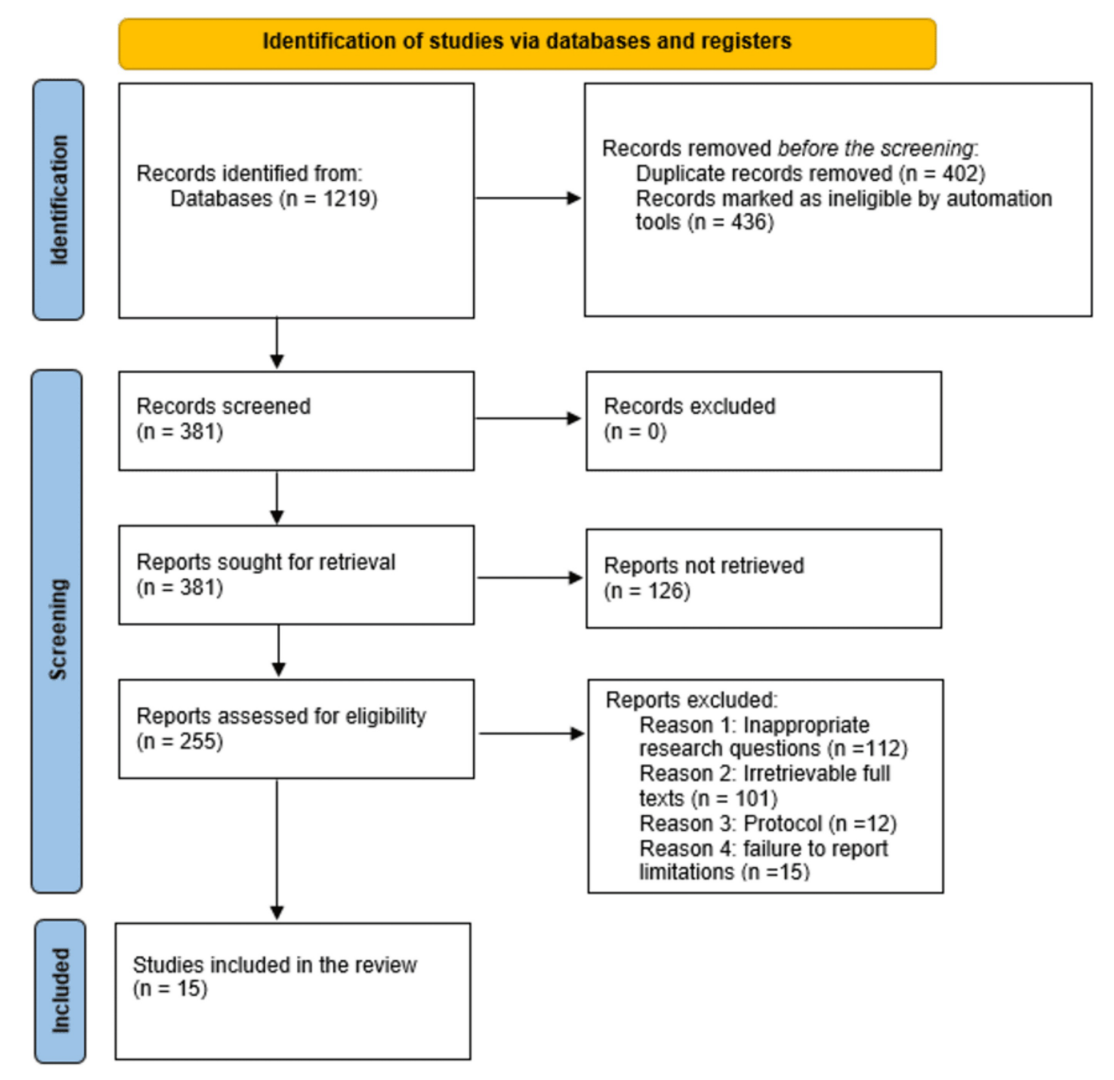
PRISMA flow diagram indicating the study selection process for this systematic review PRISMA: Preferred Reporting Items for Systematic reviews and Meta-Analyses; n: number of studies

The selection of studies and their inclusion adhered strictly to the Preferred Reporting Items for Systematic reviews and Meta-Analyses (PRISMA) guidelines [18], resulting in a thorough database search that identified 1,219 studies. Following the initial screening, 402 duplicate studies were eliminated. The next step involved reviewing the titles and abstracts of the remaining studies, which led to the exclusion of 436 studies as they were deemed ineligible. From the 381 studies that remained, efforts were made to retrieve them and assess their eligibility. Consequently, 126 studies were found to be unattainable. Thus, 255 studies were evaluated for eligibility, leading to the exclusion of 240 studies for various reasons, including unsuitable research questions (112 studies), full texts unavailable (101 studies), protocol issues (12 studies), and lack of limitation reporting (15 studies). In the end, 15 studies met the inclusion criteria and were considered along with findings from other studies that supported our results [19-67]. The flow diagram illustrating the process of study selection is presented in Figure 1.

A summary of the studies and their findings is shown in Table 1. 

**Table 1:** Summary of the studies included in this systematic review

Study/Citation	Study design	Sample characteristics	Findings
Osazuwa et al. [19]	Cross-sectional study	Antiretroviral naive AIDS patients in Benin City, Nigeria	The study screened patients for cryptococcal antigenemia and has provided important insights into the prevalence of the disease along with implications for early and effective diagnosis and management
Constance et al. [20]	Prevalence study	HIV-infected patients attending a teaching hospital in Benin, Nigeria	The study identified the prevalence rate of serum cryptococcal antigen in HIV-infected persons and stressed the importance of regular screening in high-risk populations.
Gomerep et al. [22]	Observational study	HIV-1 infected patients in North-Central Nigeria	The study established the frequency of cryptococcal meningitis, thereby indicating its significance as a major cause of morbidity in persons with HIV/AIDS.
D'Souza et al. [34]	Genomic study	*Cryptococcus gattii* isolates	The study explored genome variation in *Cryptococcus gattii*, indicating its virulence mechanisms in immunocompetent persons. It also identified genetic diversity contributing to its adaptation and pathogenicity, providing important insights into its rise as a noteworthy pathogen.
Pappas [35]	Systematic review and meta-analysis	Cryptococcosis-infected non-HIV-infected patients	The study analyzed *Cryptococcus* infections in non-HIV patients, stressing the exceptional clinical presentations and management difficulties. It also disclosed how the affected population is at risk from conditions such as immunosuppressive therapy and organ transplants.
Maziarz et al. [36]	Systematic review and meta-analysis	191 studies	The study offered a comprehensive evaluation of cryptococcosis, describing the pathogenesis, various clinical manifestations, and the extant treatment strategies. It also emphasized the significance of early diagnosis and antifungal treatment in enhancing treatment outcomes.
Mdodo et al. [37]	Antifungal susceptibility study	*Cryptococcus neoformans* isolates from AIDS patients	The study assessed the antifungal vulnerability of *Cryptococcus neoformans* among Kenyan patients with AIDS and disclosed its increased susceptibility to common antifungals. It also emphasized the need for surveillance to regularly screen for resistance trends effectively.
Yang et al. [38]	Cross-sectional study	Clinically suspected cryptococcosis cases in Jiangxi Province, South Central China	The study demonstrated a higher prevalence of HIV-related cryptococcosis in Jiangxi, China, in addition to reporting an increment in fluconazole resistance. It also underlined the importance of alternative treatment strategies.
Kassaza et al. [40]	Observational study	HIV patients in Africa	The study evaluated the genotypic diversity of *Cryptococcus neoformans* in African patients with HIV. It disclosed the existence of diverse genotypes related to varying clinical outcomes, signifying implications for customized management.
Jackson et al. [41]	Systematic review and meta-analysis	Clinical isolates of *Cryptococcus neoformans* from individuals with HIV	The study emphasized the importance of clinical isolates in studying *Cryptococcus neoformans*, highlighting how research on clinical strains improves understanding of virulence and resistance mechanisms.
Tenforde et al. [43]	Experimental study	HIV-infected persons living in developed and resource-limited settings	The study assessed the disparities in the management of HIV-related cryptococcal meningitis between the developed and resource-limited contexts, advocating for enhanced diagnostic and treatment interventions.
Chen et al. [46]	Meta-analysis	Not stated	The study explored the host-pathogen battle in *Cryptococcus neoformans* infections affecting the central nervous system, indicating the immune evasion mechanisms and the role of host response in the disease outcomes.
Mahmood et al. [50]	Systematic review and meta-analysis	116 studies	The study assessed signaling pathways in *Cryptococcus neoformans *virulence and identified pathways vital for pathogenicity and survival, highlighting potential targets for antifungal development.
Fu et al. [52]	Experimental study	Mice infected with macrophages carrying wild-type *Cryptococcus neoformans *strain	The study disclosed that *Cryptococcus neoformans* urease modulates phagolysosomal pH during intracellular pathogenesis, influencing immune responses and fungal survival.
Asemota et al. [53]	Cross-sectional study	HIV-infected individuals in Southern Nigeria	The study evaluated the hematological parameters and micronutrient levels in Nigerian patients with HIV and disclosed the existing correlations between deficiencies and immune suppression.

Discussion

Cryptococcus Species Seroprevalence in the Abuja Population

Recent research has revealed a seroprevalence of 2.40% for serum CrAg among individuals living with HIV/AIDS in Abuja [19,20]. This percentage is much lower than the seroprevalence of 12.7% observed in ART-naïve individuals with HIV/AIDS in Benin City, Nigeria, 13.5% in Kampala, Uganda, 12.9% in Bangkok, and 12.2% in the Congo [19,20]. Likewise, these study results show a lower prevalence than the 7% found in ART-naïve AIDS patients in South Africa, and 9.2% in ART-naïve HIV/AIDS patients in Thailand [20,21]. The lower rate in these studies reflects the burden of cryptococcal infection among individuals living with HIV/AIDS in FCT, Abuja. Understanding the seroprevalence rates is crucial, as this pathogen often leads to severe disease. For instance, research in Benin City has shown that the occurrence of CrAg is associated with decreased CD4 cell levels in individuals with HIV/AIDS [19]. Other studies focusing on serum CrAg prevalence in HIV/AIDS patients consistently report higher rates, especially in those with lowered CD4 cell counts [19,22]. Furthermore, investigations carried out in New York City, United States, have reported an annual prevalence rate between 6.1% and 8.5% in people living with HIV/AIDS, with no significant gender disparity (ratio 1:1) [23]. Additionally, Zhao et al. have suggested that while the overall incidence rate of cryptococcal infections is largely unknown, it is more prevalent among individuals living with HIV/AIDS in developing areas like Africa and Southeast Asia compared to developed places such as the United States. In Europe, its occurrence is notably lower [24].

In terms of factors like sex and age, female patients show a higher prevalence of seropositivity compared to male patients (2.57% vs. 2.04%) [25]. Furthermore, infection rates are notably high among sexually-active individuals between 18 to 30 years of age, with prevalence figures ranging from 2.98% to 3.06% [25]. However, no significant differences in prevalence rates were noted between genders, even though variations in distribution across different age groups were significant [25]. These findings have been supported by Gomerep et al. The study revealed a greater infection prevalence in female patients living with HIV/AIDS compared to male patients [22]. Conversely, in the United States, the prevalence of the disease is more pronounced in male patients than the females ones [26]. However, no significant difference in HIV/AIDS prevalence rates has been found between the ART-naïve female and male patients residing in Benin City (ratio of 1:1) [27]. Additionally, age seems to play a minimal role in seropositivity among individuals living with HIV/AIDS. Research by Ferreira-Paim et al. in Brazil's Amazonas state indicated that younger male patients between 16 and 30 years of age had a higher rate of cryptococcosis [28].

Concerning the relationship between epidemiology and occupation, research has shown that the occurrence of cryptococcosis was less than 1% across most occupational categories examined, with a greater prevalence among civil servants (38.53%) and traders (17.05%) [29]. Various clinical studies have reported uneven prevalence rates. This is significantly associated with how well a hospital or clinical facility can manage severe immunosuppression, such as cryptococcosis, meningitis, and HIV/AIDS [30,31]. Additionally, the results of these studies have confirmed a high burden of *Cryptococcus* in individuals living with HIV/AIDS in Abuja, Nigeria. The relatively lower incidence of cryptococcosis in FCT Abuja has been linked to the use of combination ART (cART) in many patients. Furthermore, the introduction of effective ART has led to a notable decrease in opportunistic infections related to HIV/AIDS [32]. Consequently, over 75% of cryptococcosis cases have been associated with HIV/AIDS and primarily occur in situations where the CD4 T-lymphocyte count is below 50 cells/µL [33]. A study carried out in France found that cryptococcal infections were associated with lowered CD4 counts due to the immunosuppression from HIV/AIDS [34]. Similar observations have been noted in the Pacific Northwest of the United States and British Columbia, Canada [35]. Thus, the primary risk factor for cryptococcosis has been recognized as immunosuppression caused by HIV/AIDS, and this was confirmed by Maziarz et al. [36]. Additionally, other significant invasive fungal species occurring at elevated frequencies within the *C. neoformans* blood cultures include *Aspergillus* species, *C. tropicalis*, and *Candida albicans*, with incident rates of 25.66%, 5.31%, and 6.19%, respectively [37-41]. This is above 5% and raises public health concerns as these species rank high on the epidemic ladder [42].

Susceptibility of Circulating Clinical Isolates of C. neoformans to Antifungal Drugs of Choice Used to Treat Cryptococcosis

Among the various drugs assessed, about 18.8% of the clinical isolates of *C. neoformans* showed susceptibility to voriconazole (VRZ) and itraconazole (ITZ) [37]. There is also a connection between high resistance rates and difficulties in achieving improved treatment results, particularly in HIV patients affected by cryptococcosis. Amphotericin B (AMB) is the preferred treatment option, particularly during the initial phase of therapy [36]. Research has revealed instances of resistance to AMB in patients with HIV/AIDS as well as in vitro [37,38], linked to the emergence of resistant mutants. Nonetheless, the total number of individuals who have exhibited resistance to AMB has remained low, consistent with the results in this study [39].

Numerous studies have revealed that nearly one in five *C. neoformans* isolates exhibit resistance to AMB, ketoconazole (KTZ), and fluconazole (FLZ), posing a significant public health challenge [24,4,41]. Additionally, the increased level of resistance to FLZ is particularly concerning since this medication is currently the primary maintenance treatment for individuals living with HIV/AIDS, and resistance has been reported in severely immunocompromised patients undergoing azole therapy for *C. neoformans *[41]. A heightened risk of developing resistance has been observed in patients who have received extended prophylactic treatment [42,43]. In this context, five out of 11 isolates of *C. neoformans* were susceptible to developing resistance against AMB, KTZ, and FLZ. Liu et al. also indicated that individuals with HIV/AIDS suffering from cryptococcosis had strains of *C. neoformans* that were resistant [44].

Studies have also reviewed the extracellular enzymes found in about 91% of isolates [43-45]. These enzymes were associated with the virulence and pathogenesis of the circulating *Cryptococcus* species and included DNAse (DNs), urease (URs), catalase (CTL), acid phosphatase (APPT), phospholipase B (PPB), amylase (AML), xylosylphosphotransferase (XPPt), aspartyl protease (ASPT), laccase (LCs), metalloprotease (MTLPs), phospholipase C (PPC), superoxide dismutase (SOD), and protease (PRT) [43-45].

Cryptococcal extracellular proteins are recognized as important esterases. Other pathogens have been associated with lipase and proteinase in relation to their virulence [44-46]. A study by Almeida et al. revealed that *C. neoformans* produces an extracellular phospholipase that can significantly disrupt mammalian cell membranes and facilitate the invasion of host tissues. In a similar manner, research by Chen et al. indicated that the PRTs from *C. neoformans *play a crucial role in virulence by initiating an invasion of the host tissues [45]. An analysis of the studies included in this systematic review has demonstrated that the clinical strains showed significant enzymatic activity, indicating a positive detection of these enzymes [45-48]. For example, APPT is an enzyme linked to virulence in *Galleria mellonella* and in mouse models [45-48]. Additionally, mannosyltransferase enzymes, which are involved in capsule formation and other fungal virulence factors, like smaller extracellular vesicles and increased cell sizes, have a significant effect on the expression of various characteristics of virulence [45-48]. Changes to these capsular structures during murine infections can enhance virulence.

In *C. neoformans*, mannosyltransferase is crucial for synthesizing the capsule, particularly in serotype A. It influences endothelial crossing in vitro as well as virulence in murine models [45,49]. LCs, which is a glycosylated copper enzyme that synthesizes melanin and oxidizes diphenolic substrates such as catecholamines, also plays a significant role in virulence by aiding in melanin production [50]. Melanin functions as an antioxidant and protects the *C. neoformans* cells from oxidative damage while also enhancing resistance to various antifungal treatments [50].

It is important to note that *C. neoformans* generates DNs and PRT that enhance its virulence by breaking down host tissues, interfering with immune responses, and providing nucleotides that support the infection [43-45]. Research has demonstrated a strong link between DNs production and URs activity. Clinical strains show increasingly pronounced DNs activity, highlighting its crucial role as a virulence factor [43]. These enzymes work to cause tissue damage, improve fungal survival, and increase pathogenicity. URs, which is present in nearly all isolates of *C. neoformans* (99.6%), aids in the conversion of urea into carbamate and ammonia, establishing it as a significant virulence factor [46]. Several studies indicate that disrupting the URs gene significantly diminishes the pathogen's virulence in vitro [43,45,48].

In addition, the SODs found in *C. neoformans *contribute to its virulence by promoting growth within macrophages and offering protection against the superoxide generated by the host [51]. Phospholipases, including PPB and PPC, play a role in degrading cell membranes and facilitating the adhesion of the fungus to host cells [51]. PPB assists in tissue invasion by breaking down phospholipids in the lung and plasma membranes, while PPC helps produce melanin, build resistance to antifungal agents, and maintain cell wall integrity [45]. PRTs further boost tissue invasion and the ability to penetrate host cells, which is vital for virulence [45,49].

Micronutrients, Cryptococcus, and HIV/AIDS

Moderate correlations have been observed between serum zinc levels and CD4 cell counts, with significant zinc deficiency noted at various stages of illness among individuals living with HIV/AIDS [52,53]. Compared to healthy individuals, lower zinc levels (hypozincemia) were seen in individuals living with HIV/AIDS, irrespective of cryptococcosis [53]. The impact of cryptococcosis on zinc levels has been documented at different immunological stages. For example, phase I recorded 83.21 µg/dL, phase II noted 97.09 µg/dL, and phase III measured 74.15 µg/dL [53]. These findings align with the results from numerous global studies on HIV/AIDS. Additionally, the notable decreases in micronutrient levels during the progression of HIV/AIDS further reinforce the connections between immunosuppression seen in this condition and micronutrient deficiencies [53,54].

This systematic review further establishes the direct correlation between serum zinc levels and CD4 cell counts. A decline in CD4 cell counts has been associated with a reduction in serum zinc levels. Multiple studies have revealed significant zinc deficiencies in individuals diagnosed with HIV/AIDS at various stages of the disease [53-56]. Zinc deficiency appears to be increasingly pronounced in those affected by HIV/AIDS and cryptococcal infections [56]. However, several investigations have concluded that zinc deficiency is not a contributing factor to HIV/AIDS, or vice versa [56]. These observations could be due to the heightened demand for zinc, given that the essential HIV nucleocapsid and integrase protein (integral to the assembly of the infectious virus) utilize zinc fingers, which need zinc for their functionality and structural integrity [57-62]. This finding aligns with those of previous studies that have reported higher serum zinc levels in persons living with HIV. Nevertheless, variations in dietary zinc intake from natural sources is likely to contribute to the observed differences in serum zinc levels across individuals [53,56,63]. This explanation is consistent with the conclusions of earlier research.

In this study, we observed that in stage I of asymptomatic HIV disease, serum copper (Cu) levels were elevated compared to stages II and III. Serum Cu levels and the severity of HIV/AIDS disease were inversely correlated. This aligns with previous findings regarding Cu deficiency among individuals with differing HIV/AIDS status and stages of cryptococcal disease. Numerous studies highlighted in this systematic review have identified associations between micronutrient levels and HIV/AIDS, particularly in relation to immunological status and disease progression. Asemota et al. reported significantly higher serum Cu levels in people living with HIV/AIDS and suffering from opportunistic infections [53]. This finding contrasts with observations made by Meidani et al. [60] and Pennap et al. [61], who noted no differences in serum Cu levels between those living with HIV/AIDS and healthy individuals. Nevertheless, our study revealed an inverse correlation with serum Cu levels. While elevated serum Cu levels are often present in inflammatory and infection states, this phenomenon can be attributed to increased hepatic synthesis and ceruloplasmin release.

Tenforde et al. [43] revealed that the average serum selenium (Se) level among HIV/AIDS patients infected with *Cryptococcus* is notably low, with severe deficiencies particularly observed in patients at Stage III. In a majority of studies, severe Se deficiency has been documented during the advanced stages of HIV/AIDS and *Cryptococcus* disease, demonstrating a significant correlation with CD4 cell count [20,55,59,63]. This suggests that various factors associated with immune deficiency play crucial roles in determining Se levels in patients with *Cryptococcus *and HIV/AIDS. Our findings are consistent with those of previous studies, which have shown that deficiencies in different trace elements contribute to the effects of malnutrition on immune function and anemia development [44-47,52,55]. Furthermore, alterations in serum trace element levels have been identified in instances of HIV co-infections and tuberculosis [55,59,63].

Numerous studies have utilized the plasma Cu/zinc ratio in the clinical assessment of zinc deficiency, particularly in cases of severe disease [56-60]. In a notable study involving over 100 gay males living with HIV/AIDS, a Cu/zinc ratio greater than one was associated with increased mortality rates [63], underscoring its significance in predicting survival and disease progression in these patients. Furthermore, no significant correlation has been observed between serum Se levels and age or sex. Se deficiency has been implicated in the progression of and mortality due to HIV/AIDS. In alignment with our study, several others have reported diminished serum Se levels among people living with HIV/AIDS [64,65]. However, it is noteworthy that severe Se deficiency predominantly occurs in the latter stages of HIV/AIDS and is typically absent in asymptomatic patients. Additionally, there is a strong correlation between the CD4 cell count and serum Se levels. Moreover, several prior investigations have indicated that in these patients, Se deficiency remained independent of factors such as malabsorption, serum Se levels, and CD4 cell count when it concerns mortality and opportunistic infections [55]. The connection between *C. neoformans*, opportunistic infections, and the widely expressed virulence markers is recognized as the hallmark of HIV infection and its subsequent progression to AIDS. This progression involves the gradual depletion of CD4+ T cells and correlates with the incremental impairment of cellular immunity along with an increased susceptibility to various opportunistic infections [66].

Regarding its molecular characterization, successful amplification occurred in seven isolates, which confirmed the presence of *C. neoformans* with a genomic prevalence rate of 0.93% [38]. The suitability of polymerase chain reaction (PCR) analysis has been further demonstrated by its capability to effectively distinguish between closely-related isolates within specific geographical regions [33]. This underscores the relatively infrequent and low incidence rate of *C. neoformans* within the populations reviewed in the literature. Contributing factors to the observed lower prevalence rates may include the administration of anti-HIV/AIDS therapies and cART, which inadvertently influence the microbial pathogen levels [67]. Moreover, other studies indicate that medications such as indinavir and oseltamivir exhibit inhibitory effects on both the influenza virus neuraminidase and HIV PRT. This suggests that their interactions with PRT and enzymatic inhibitors are likely to have a beneficial impact on *C. neoformans* infections, particularly in cases where these treatments have been associated with in vitro fungal proliferation and growth inhibition [45]. This review has revealed two distinct fingerprint patterns (identified by pertinent short-sequence primers) in clinical isolates of *C. neoformans, *particularly from studies conducted in Abuja, Nigeria [19,20,22,53].

Study limitations

Given that this study is a systematic review, a major limitation is the potential for selection and publication biases. Further, variations in the designs of the included studies, their methodologies, and sample sizes limit the generalizability of the findings. Further, this secondary data might additionally introduce heterogeneity in the outcome measures, which makes a direct comparison difficult.

## Conclusions

The low prevalence of* C. neoformans* is linked to its rarity and the impact of medications like indinavir and oseltamivir used in cART, which inhibit fungal growth by targeting PRTs. The virulence of *C. neoformans* relies on specific secreted enzymes correlated with its pathogenesis. Future research should focus on these enzymes and the enzymatic targets of the antifungal agents. Zinc deficiency is prevalent among people living with HIV/AIDS on ART, indicating that zinc supplements are necessary for better management. Additionally, Se depletion increases the risk of opportunistic infections in these patients. Monitoring antifungal susceptibility is crucial given the rising resistance rates of *C. neoformans,* especially for selecting effective treatments. Current therapies include AMB, ITZ, and VTZ, while FLZ has been used for prophylaxis. The prevalence of significant antifungal resistance in Abuja, Nigeria, highlights the need for ongoing surveillance and intervention strategies to control fungal infections in people living with HIV/AIDS.
